# Eight New Genomes and Synthetic Controls Increase the Accessibility of Rapid Melt-MAMA SNP Typing of *Coxiella burnetii*

**DOI:** 10.1371/journal.pone.0085417

**Published:** 2014-01-21

**Authors:** Edvin Karlsson, Anna Macellaro, Mona Byström, Mats Forsman, Dimitrios Frangoulidis, Ingmar Janse, Pär Larsson, Petter Lindgren, Caroline Öhrman, Bart van Rotterdam, Andreas Sjödin, Kerstin Myrtennäs

**Affiliations:** 1 CBRN Defence and Security, Swedish Defence Research Agency, Umeå, Sweden; 2 Bundeswehr Institute of Microbiology, Munich, Germany; 3 National Institute for Public Health and the Environment, Bilthoven, the Netherlands; Texas A&M Health Science Center, United States of America

## Abstract

The case rate of Q fever in Europe has increased dramatically in recent years, mainly because of an epidemic in the Netherlands in 2009. Consequently, there is a need for more extensive genetic characterization of the disease agent *Coxiella burnetii* in order to better understand the epidemiology and spread of this disease. Genome reference data are essential for this purpose, but only thirteen genome sequences are currently available. Current methods for typing *C. burnetii* are criticized for having problems in comparing results across laboratories, require the use of genomic control DNA, and/or rely on markers in highly variable regions. We developed in this work a method for single nucleotide polymorphism (SNP) typing of *C. burnetii* isolates and tissue samples based on new assays targeting ten phylogenetically stable synonymous canonical SNPs (canSNPs). These canSNPs represent previously known phylogenetic branches and were here identified from sequence comparisons of twenty-one *C. burnetii* genomes, eight of which were sequenced in this work. Importantly, synthetic control templates were developed, to make the method useful to laboratories lacking genomic control DNA. An analysis of twenty-one *C. burnetii* genomes confirmed that the species exhibits high sequence identity. Most of its SNPs (7,493/7,559 shared by >1 genome) follow a clonal inheritance pattern and are therefore stable phylogenetic typing markers. The assays were validated using twenty-six genetically diverse *C. burnetii* isolates and three tissue samples from small ruminants infected during the epidemic in the Netherlands. Each sample was assigned to a clade. Synthetic controls (vector and PCR amplified) gave identical results compared to the corresponding genomic controls and are viable alternatives to genomic DNA. The results from the described method indicate that it could be useful for cheap and rapid disease source tracking at non-specialized laboratories, which requires accurate genotyping, assay accessibility and inter-laboratory comparisons.

## Introduction

*Coxiella burnetii* is the etiological agent of Q fever. In recent years, its prevalence in both humans and animals has increased significantly in Europe [1]–[7]. In particular, the epidemic in the Netherlands in 2009 left almost 4,000 patients diagnosed with this disease, has highlighted the demand for improved more selective tools for its diagnosis and for the typing of *C. burnetii*, based on methods that have high levels of inter-laboratory reproducibility and that can be performed at non-specialized laboratories (Fig. S1) [2].

*C. burnetii* is an obligate intracellular bacterium. Human infections typically arise via the inhalation of air-borne bacteria emitted from infected animals such as goats or sheep, but many other routes of infections and sources have been described [8]–[10]. Because of its high infectivity and extreme resistance to environmental stress factors among other things, the CDC has identified *C. burnetii* as a category B potential bioterrorism agent [11]. Exposure to this bacterium results in the development of a subclinical disease in approximately 60% of all cases, while 40% of exposed individuals develop a mild flu-like disease known as acute Q fever. Patients who develop pneumonia may require hospitalization, and primary infections can in some cases progress to chronic Q fever and Q fever fatigue syndrome [12].

Q fever is generally diagnosed on the basis of serological analyses. However, serological seroconversion is usually delayed 7 to 15 days after onset of clinical symptoms [13]. Therefore, real-time PCR analyses of serum samples are indispensable for early diagnosis of acute Q fever [14], [15]. PCR methods targeting the multi-copy IS1111 element [15], [16] and highly conserved genes such as the single copy outer membrane-associated com1 protein [17], [18] have therefore been developed. A number of typing methods have been used to analyze the genetic diversity among *C. burnetii* isolates, including pulsed-field gel electrophoresis (PFGE) [19], restriction fragment length polymorphism (RFLP) [20], microarray methods [21], [22], multiple-locus variable-number tandem-repeats analysis (MLVA) [2], [23]–[25], IS1111 [15], [16], multispacer sequence typing (MST) [26], and SNP typing [27], [28]. Results from these publications and analysis of the published *C. burnetii* genomes have shown that this bacterium has a highly clonal population structure [28]. Moreover, samples obtained from sheep, goats and humans infected during recent outbreaks in the Netherlands exhibit low genetic diversity and a single dominant genotype. This has prevented accurate source tracking [2], [25], [29] and the establishment of a direct relationship between isolates from infected humans and animals during these outbreaks. At present, *C. burnetii* is usually typed using MLVA assays, which target variable number tandem repeats. This technique increases the typing resolution, but the results obtained are not easy to use in inter-laboratory comparisons of typing results, primarily due to difficulties in identifying the correct number of tandem repeats [30]. In addition, unrelated strains may have identical numbers of tandem repeats in MLVA assays (a phenomenon known as homoplasy). Together, these factors introduce considerable uncertainty when trying to identify the true genetic relationships between isolates. Sequencing of the MLVA loci could be useful in obtaining accurate estimates of repeat numbers, as demonstrated by Bruin *et al*. [25]. However, this does only partially address issues arising from inter-laboratory differences in MLVA results due to the remaining differences in the scoring of repeats, which would have to be resolved by establishing a detailed consensus regarding repeat sequences and allowable variation.

A recently developed typing strategy [28], which also has been used to type other highly clonal bacteria as *Francisella tularensis* [31]–[35], *Yersinia pestis* [36], [37], and *Bacillus anthracis* [38], is based on the identification of canonical single nucleotide polymorphisms (canSNPs), *i.e.* each canSNP is specific to a branch of a phylogenetic tree. The canSNPs are easy to decipher and provides highly accurate phylogenetic data due to a reduced likelihood of homoplasy. Data obtained in different laboratories are therefore easy to compare. Hornstra *et al.* [28] examined canSNPs located inside the MST sequence regions of *C. burnetii*, and determined their SNP status using a method known as Melt-MAMA (mismatch amplification mutation assay, coupled with melt analysis). In principle, any SNP detection method that allows for unambiguous identification could be used for the same purpose; other potentially suitable techniques include hydrolysis probe assays [27], [28] and sequencing [26]. Phylogenetic relationships between strains at the desired typing level are easily established using the canSNP DNA signatures obtained using this method. However, the use of canSNPs in highly variable regions, such as intergenic MST regions, presents the potential for unidentified variation to impede primer binding and subsequent amplification using PCR.

In principle, typing could be performed based on canSNPs in conserved regions of the core genome as a complement to variable MST regions. The viability of this approach is currently limited by the fact that only thirteen *C. burnetii* genomes have been published to date (Table 1) [22], [39], [40]. This restricts the diversity and resolution of the SNP markers that can be identified. However, a rapid increase in the number of available genome sequences for *C. burnetii* can be anticipated in the near future as a consequence of recent technical advances in genome sequencing [41] and methods for culturing bacterial samples [42]. Meanwhile and in cases when highest possible typing resolution is needed, signatures that are not ideal for assay development such as SNPs in MST regions may be selected for canSNP assays because of a lack of alternative loci in providing resolution on very short branches. In this work, we report the sequencing of the genomes of eight additional *C. burnetii* isolates. The sequencing data were used to conduct phylogenetic analyses for this species and to identify canonical SNPs in intragenic conserved regions of the chromosomal core genome. The selected canSNPs were then used to develop Melt-MAMA assays for typing of *C. burnetii*. One limitation of PCR based SNP assays is that they require the availability of genomic control DNA when determining SNP status; this is not readily accessible to non-specialized laboratories, mainly due to the fastidious growth conditions for *Coxiella*, associated with high costs for specialized equipment and trained personnel. PCR-amplified control vectors have been used to circumvent this issue in studies on *Francisella* [43], while synthetic vectors have been used in studies on other bacteria [44]. Therefore, to make the assays accessible to laboratories that lack control DNA, we developed synthetic control templates. Finally, the performance of the new typing assays was evaluated using DNA extracted from twenty-six isolates and three infected placenta tissue samples.

**Table 1:** The 21 genomes, 26 isolates and three tissue samples analyzed in this study.

Strain/Sample	Alt. name	Plasmid	Origin	Year	Accession no.	DNA source^a^	DNA amplification^b^
Ohio	ATCC VR 542	QpH1	Milk, USA	1955	PRJNA197125^c^	ATCC	no
Nine Mile	RSA493	QpH1	Tick, USA	1935	AE016828.2	WSU	no
S4^d^		QpH1	Sheep, Sweden	1990	PRJNA197126^e^	JLU/FOI^f^	no
Australian QD	RSA425	QpH1	Human blood, Australia	1939	SRS213928	N/A	
C2^g^		QpH1	Hay, Sweden	1997	PRJNA197120^c^	FOI	no
Cb175		QpH1	Guyana		PRJNA199747	N/A	
Henzerling	RSA331	QpH1	Human, Italy	1945	CP000890.1	N/A	
Innsbruck	Z2534	QpH1	Goat, Austria		PRJNA197122^h^	JLU	no
2338		QpH1	Cow, Germany	2003	PRJNA197123^e^	IMB	MDA
Z349-36/94		QpH1	Sheep,Germany	1994	PRJNA197128^e^	IMB	no
M44	RSA459, Grita	QpH1	Human blood, Italy	1945	SRS291555	N/A	
Cb109		QpH1	Human, Germany		AKYP00000000	N/A	
Z3055		QpH1	the Netherlands		PRJNA199725	N/A	
Dugway	5J108-111	QpDG	Rodent, USA		CP000733.1	N/A	
Q321		QpDV	Cow's milk, Russia		AAYJ00000000	N/A	
CbuK	Q154, K	QpRS	Human, USA	1976	CP001020.1	N/A	
Priscilla	Q177	QpRS	Goat, USA	1980	AAUP00000000	IMB	MDA
Namibia		QpRS	Goat, Namibia	1991	PRJNA197124^i^	IMB	MDA
CbuG	Q212	Integrated	Human, Canada		CP001019.1	N/A	
McMaster	Q172	Integrated	Human placenta		SRS213929	N/A	
Scurry	Q217	Integrated	Human, USA	1981	PRJNA197127^c^	WSU	no
Brasov		QpH1	Human, Romania		N/A	IMB	no/MDA
CS-KL 8		QpH1	Tick, Slovak Republic	1989	N/A	IMB	MDA
J-3		QpH1	Cow, Japan		N/A	IMB	MDA
Utvinis		QpH1	Human, Romania		N/A	JLU	no/MDA
Balaceanu		QpH1	Human, Romania		N/A	JLU	no
Geier		QpH1	Human, Romania		N/A	IMB	MDA
CS-Florian		QpH1	Human blood, Slovak Republic	1956	N/A	IMB	MDA
Soyta		QpH1	Cow, Switzerland	1965	N/A	IMB	MDA
Herzberg		QpH1	Human, Greece	1946	N/A	FOI/IMB^j^	no/MDA
Andelfingen		QpH1	Cow, Switzerland	1965	N/A	IMB	MDA
München		QpH1	Sheep, Germany	1969	N/A	FOI	no/MDA
CS-Ixodes		QpH1	Tick, Russia	1962	N/A	IMB	MDA
RT-I		QpH1	Mice, Russia	1958	N/A	IMB	MDA
S1		QpH1	Sheep, Sweden	1990	N/A	IMB	MDA
F-4		QpDV	Human, France	1978	N/A	IMB	MDA
F-1		QpRS	Human, France	1978	N/A	IMB	MDA
03784^k^		QpH1	Sheep placenta, Noord-Brabant, the Netherlands	2009	N/A	RIVM	no
01040^k^		QpH1	Goat placenta, Noord-Brabant, the Netherlands	2007	N/A	RIVM	no
01050^k^		QpH1	Goat placenta,Limburg, the Netherlands	2007	N/A	RIVM	no

^a^ Received from WSU = Washington State University, USA; FOI = Swedish Defence Research Agency, Umeå, Sweden; JLU = Justus-Liebig University, Giessen, Germany; IMB = Bundeswehr Institute of Microbiology, Munich, Germany; ATCC = American Type Culture Collection, USA; RIVM = National Institute for Public Health and the Environment, Utrecht, the Netherlands.

^b^ no = no amplification; MDA = multiple displacement amplification; no/MDA = no amplification used for SNP assay, MDA used for genome sequencing.

^c^ 36 bp single-end reads (Illumina). Mean insert size not applicable.

^d^ Sampled by FOI. Isolated by JLU.

^e^ 100 bp paired-end reads (Illumina). Mean insert size (standard deviation): S4; 322(145), Z349-36/94; 269(97), 2338; 478(31).

^f^ Isolated by JLU. Cultured by FOI.

^g^ Isolated by FOI [54].

^h^ 76 bp single-end reads (Illumina). Mean insert size not applicable.

^i^ 150 bp paired-end reads (Illumina). Mean insert size (standard deviation): Namibia; 297(92).

^j^ Genomic DNA was prepared on Herzberg isolate at FOI, MDA DNA was prepared from the Herzberg isolate at IMB.

^k^ ID at RIVM.

## Results

### Genome sequencing

We sequenced the genomes of 8 *C. burnetii* isolates, increasing the number of publically available genomes for this species to 21 (Table 1, Table 2). The access to more genome sequences increased the flexibility to select signatures in general, and particularly in this work it facilitated identification of phylogenetic stable synonymous canSNPs and regions optimal for primer design for use in newly-developed typing assays (Table 3).

**Table 2:** Genome sequence data statistics of the eight new *Coxiella burnetti* strains sequenced in this study.

Genome	Mean sequence depth	Number of positions with more than 10× coverage	Reference genome length^a^	Percentage of reference genome^a^ with more than 10× coverage	Percentage of filtered reads aligning to all published *Coxiella*	Total number of reads^b^
C2	438	1,968,972	1,995,281	98,7%	95,6%	24,693,231
Z2534, Innsbruck	86	1,974,783	1,995,281	99,0%	96,9%	2,593,982
ATCC VR 542, Ohio	275	1,995,169	1,995,281	100,0%	90,2%	16,274,215
Q217, Scurry	157	1,942,040	1,995,281	97,3%	90,4%	9,518,013
Namibia	87	1,956,716	1,995,281	98,1%	24,0%	5,462,726^c^
2338	113	1,969,807	1,995,281	98,7%	20,9%	11,434,898^c^
S4	1063	1,995,281	1,995,281	100,0%	96,7%	22,369,452^c^
Z349-36/94	924	1,975,322	1,995,281	99,0%	72,6%	24,952,742^c^

^a^ Nine Mile RSA493 (GenBank AE016828.2).

^b^ Filtered by aligning reads to *Cholocebus aethiops* draft genome (NCBI BioProject PRJNA168621).

^c^ Paired end sequences.

**Table 3:** CanSNPs and Melt-MAMA primers.

CanSNP	SNP position in Nine Mile RSA493 (GenBank AE016828.2)	Gene	Base	Primer	Primer sequence^b^ (5′-3′)	Conc. (nM)	Product Tm (°C)
C.1	1,811,671	*ponA*	C	Anc^a^	GCAATCAAACCATCTCGCtAC	200	75,4
			G	Der^a^	ccccgccccgcccgcCAATCAAACCATCTCGCcAG	200	80,3
				Com	AATGTTTATACGACGATTTCTTC	200	
C.3	559,844^c^	*lpxD*	T	Anc	AAACTAATTTCACCTGGTcGA	200	75,1
			C	Der	ggggcggggcggggcAAACTAATTTCACCTGGTaGG	200	80,6
				Com	ATTCACAATGTTGCTGCTAT	200	
C.4	604,402^c^	*dnaZX*	G	Anc	CTCGATGTTTCCAAGAAGTCcTC	1000	78,0
			A	Der	ggggcggggcggggtCTCGATGTTTCCAAGAAGTCgTT	200	82,4
				Com	GTTGCTTAAAGCCCGTAC	200	
C.5	847,839	*dedE*	T	Anc	GCTGTTTTATTGTTGTTCGTTgGT	200	74,5
			C	Der	ccccgccccgcccctCTGTTTTATTGTTGTTCGTTcGC	200	79,6
				Com	GCAATCAAACTCCCATCTT	200	
C.6	648,943	*CBU0700*	G	Anc	GTTCGTAGGGATCGCTAcTG	800	76,1
			A	Der	ccccgccccgccccGGTTCGTAGGGATCGCTAgTA	200	79,8
				Com	ccccgcccgAAGCTCGAATGGGTATAATCA	200	
C.7	1,177,930^c^	*qseC*	G	Anc	ggggcggggcggggcTGGTCAGTCGAAAATGGGAAaAC	200	80,8
			A	Der	TGGTCAGTCGAAAATGGGAAtAT	800	75,5
				Com	CTTATTCAACGCAATCTCCTAA	200	
C.8	1,356,018	*sdhA*	C	Anc	CTCGCAACACCATACCAcTC	600	75,5
			T	Der	ggggcgggcggggtCTCGCAACACCATACCAgTT	200	80,2
				Com	CAACCAGTAACGCTTATACC	200	
C.9	33,004^c^	*fabA*	G	Anc	AACCTGGTTGCTTAGGCtTC	800	74,0
			A	Der	ggggcggggcgggttAACCTGGTTGCTTAGGCaTT	200	78,6
				Com	GAGATATATAACCCCAATAATTGC	200	
C.10	1,436,254	*CBU1483*	C	Anc	CAATACCCAACATGCCAAAAtTC	800	76,2
			T	Der	ggggcggggcggcgCAATACCCAACATGCCAAAAgTT	200	80,9
				Com	TGGGATTGGCTTTCATTGT	200	
C.11	1,792,411^c^	*CBU1863*	G	Anc	TCCCAACAAATGGACAGGTaAC	400	71,8
			A	Der	ccccgccccgccccgCCCAACAAATGGACAGGTcAT	200	76,7
				Com	CCCAACTTGAAACGCTTT	200	
C.6 Cont			A	Der Cont	TGGTTCGTAGGGATCGCTAATA	200	
				Com Cont	AAAGCTCGAATGGGTATAATCAAA	200	

^a^ SNP state could not be determined whether ancestral or derived.

^b^ Antepenultimate mismatches and primer-tails are presented in lower case letters.

^c^ Primers designed on the reverse complement strand to Nine Mile RSA493.

### Phylogenetic analysis

**Figure 1:**
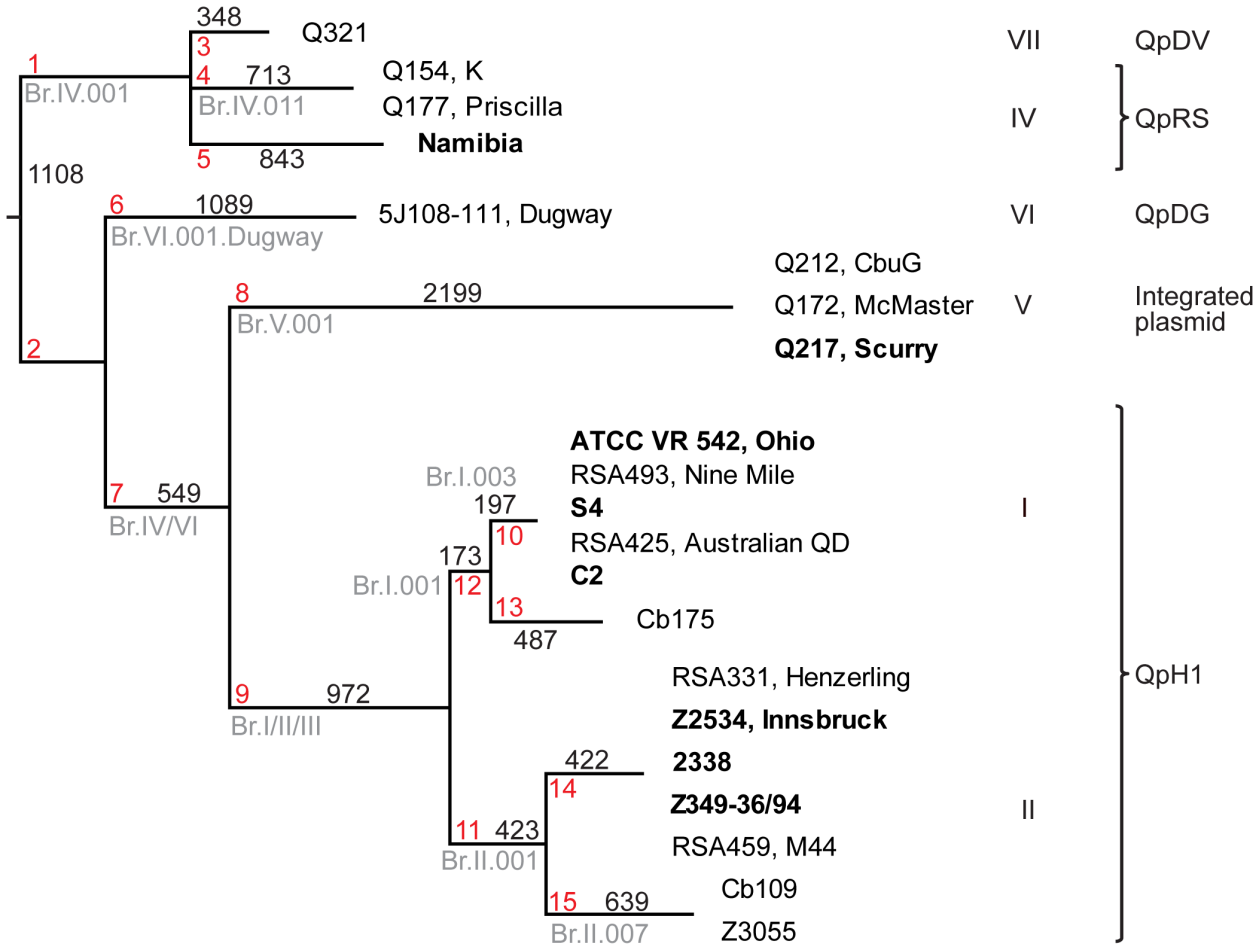
Phylogenetic tree. Branch lengths are proportional to the total number of SNPs, which are indicated above or below the branches. Genomes sequenced in this study are in bold face. The genomic groups (I–VII) and plasmid types are denoted as described [21], [26], [28]. CanSNP markers are depicted in red (C.1–C.15) and the corresponding MST markers are depicted in grey. The genomes Cb109, Cb175 and Z3055 became available after the time of assay design resulting in four new branches (C.12 to C.15). Although not included in this work, it demonstrates that the systematics of canSNPs easily can be extended when new genomes become available. The ancestral and derived state for C.1 and C.2 could not be determined due to the lack of sequence from a near neighbor (a root). Therefore, we could only design one assay C.1 that could be on either the C.1 or C.2 branches.

A total of 21 *Coxiella burnetii* genomes were included in the neighbor joining tree: all 13 publically available genomes and the 8 new genomes sequenced by us, which were selected to expand the available coverage of the genomic diversity within the species (Table 1, Fig. 1). There were a total of 1,362,088 nucleotides in common between the 21 genomes in the final dataset. The species proved to have substantial sequence identity: 10,669 SNPs were identified within the 1.36 Mbp in common between the genomes. Most of the SNPs (7,493/7,559) found in more than 1 of the genome sequences were consistent with the tree structure (Fig. 1). Consequently, only 66/7,559 SNPs were found to be homoplastic, suggesting a genetically clonal population structure. These homoplasies did not affect the assay design and were not analyzed further. Among the 3,110 sequence-unique SNPs, the majority (2,869) were unique to the Dugway (1,089), Namibia (843), Cb175 (487), Q321 (348), and Cb109 (102) strains, respectively. The overall structure of the tree based on genome sequences matched that of the previously published tree based on multispacer sequence typing (MST) [26], [28]. An *in silico* analysis of 17 previously published MLVA loci to analyze the frequency of homoplasy among these markers which could lead to incorrect interpretation of phylogenetic relationships, showed that in addition to variable number tandem repeats, these regions also contain other genetic markers such as INDELs (insertion deletion polymorphisms) (data not shown), and that the seven microsatellite (MS) loci MS01, MS12, MS22, MS26, MS27, MS31, and MS34 are incongruent with the SNP phylogeny (Table S1) [2], [23].

### A typing scheme based on synonymous canonical SNPs

A typing scheme was developed that consisted of 10 synonymous canSNPs in conserved regions containing no additional variation in the 21 genomes, each of which represented a major phylogenetic branch of *C. burnetii* previously identified by others [26], [28] (Table 3, Table 4, Fig. 1). Our canSNPs in stable regions could be used as an alternative to assayed canSNPs in the more variable MST regions in Hornstra *et al.* and complement them in two cases not assayed in [28]; C.3 targets the clade starting with branch Br.IV.003 (ST1–4); and C.5 targets the clade starting with branch Br.IV.010 (ST30). Polymorphisms and homoplasies in the MST regions were reported by Hornstra *et al.* [28]. A comparison with the genomes reported here revealed additional polymorphisms, and confirmed others. One SNP, previously identified as Cox22bp118, was located within the reverse primer site of assay Cox22bp91 (Br.I/II/III) for Q154 (CbuK, K) and Q177 (Priscilla). Also, the reverse primer position for assay Cox5bp109 (Br.III.001) was located on a 12 bp tandem repeat causing mispriming for Q154 (CbuK, K) and Q177 (Priscilla), which possibly could impede PCR amplification. One new polymorphism, representing a novel ST, was also identified within the MST SNP assay amplicon regions: the assay Cox20bp155 (Br.VI.001) includes one 1 bp INDEL in the genome of Cb175 (Br.II.002) at position 139 in Cox20 that was not previously identified in Glazunova *et al.* or Hornstra *et al.* [26], [28]. However, these additional polymorphisms need to be confirmed by resequencing.

**Table 4:** Typing results for genomes and isolates, including MST data for comparison.

Strain (genome sequenced)	Plasmid	Genomic group	C.1	C.3	C.4	C.5	C.6	C.7	C.8	C.9	C.10	C.11	Derived for canSNP	Derived for MST SNP^c^	MST branch^c^	ST^c^	CanSNP typing of non-sequenced isolates
Q321	QpDV	VII	G	**C**	G	T	G	G	C	G	C	G	C.3^b^	Cox18bp34	Br.IV.001	1–7,30	**Derived for C.3**
Q154, CbuK, K	QpRS	IV	G	T	**A**	T	G	G	C	G	C	G	C.4	Cox51bp67	Br.IV.015	8	F-1
Q177, Priscilla	QpRS	IV	G	T	**A**	T	G	G	C	G	C	G	C.4	Cox51bp67	Br.IV.015	8	F-4
Namibia^a^	QpRS	IV	G	T	G	**C**	G	G	C	G	C	G	C.5^b^	Cox18bp34	Br.IV.001	1–7,30	**Derived for C.10**
5J108-111, Dugway	QpDG	VI	C	T	G	T	**A**	G	C	G	C	G	C.6	Cox20bp155	Br.VI.001	NA	Balaceanu
Q212, CbuG	Integrated	V	C	T	G	T	G	**A**	**T**	G	C	G	C.8	Cox5bp81	Br.V.001	21	Brasov
Q172, McMaster	Integrated	V	C	T	G	T	G	**A**	**T**	G	C	G	C.8	Cox5bp81	Br.V.001	21	CS-KL8
Q217, Scurry^a^	Integrated	V	C	T	G	T	G	**A**	**T**	G	C	G	C.8	Cox5bp81	Br.V.001	21	J-3
ATCC VR 542, Ohio^a^	QpH1	I	C	T	G	T	G	**A**	C	**A**	**T**	G	C.10	Cox51bp356	Br.I.003	16,26	Utvinis
RSA493, Nine Mile	QpH1	I	C	T	G	T	G	**A**	C	**A**	**T**	G	C.10	Cox51bp356	Br.I.003	16,26	**Derived for C.11**
S4^a^	QpH1	I	C	T	G	T	G	**A**	C	**A**	**T**	G	C.10	Cox51bp356	Br.I.003	16,26	CS-Florian
RSA425, Australian QD	QpH1	I	C	T	G	T	G	**A**	C	**A**	**T**	G	C.10	Cox51bp356	Br.I.003	16,26	München
C2^a^	QpH1	I	C	T	G	T	G	**A**	C	**A**	**T**	G	C.10	Cox51bp356	Br.I.003	16,26	Soyta
Cb175^d^^,^ e	QpH1	I	C	T	G	T	G	**A**	C	**A**	C	G	C.9	Cox18bp376	Br.I.001	16,17, 26	Herzberg
RSA331, Henzerling	QpH1	II	C	T	G	T	G	**A**	C	**A**	C	**A**	C.11	Cox18bp166	Br.II.001	18,22,23,25,29	Andelfingen
Z2534, Innsbruck^a^	QpH1	II	C	T	G	T	G	**A**	C	**A**	C	**A**	C.11	Cox18bp166	Br.II.001	18,22,23,25,29	S1
2338^a^	QpH1	II	C	T	G	T	G	**A**	C	**A**	C	**A**	C.11	Cox18bp166	Br.II.001	18,22,23,25,29	CS-Ixodes
Z349-36/94^a^	QpH1	II	C	T	G	T	G	**A**	C	**A**	C	**A**	C.11	Cox18bp166	Br.II.001	18,22,23,25,29	Geier, RT-1
RSA459, M44	QpH1	II	C	T	G	T	G	**A**	C	**A**	C	**A**	C.11	Cox18bp166	Br.II.001	18,22,23,25,29	SZ2009-03784
Cb109^e^	QpH1	II	C	T	G	T	G	**A**	C	**A**	C	**A**	C.11	Cox51bp492	Br.II.007	11–15,24,32–34	SZ2007-01040
Z3055^e^	QpH1	II	C	T	G	T	G	**A**	C	**A**	C	**A**	C.11	Cox51bp492	Br.II.007	11–15,24,32–34	SZ2007-01050

^a^ Sequenced in this study.

^b^ The assays for C.3 and C.5 have no corresponding assay in Hornstra *et al.* [28]. C.3 and C.5 targets the clades starting with branches Br.IV.003 (ST1–4), and Br.IV.010 (ST30), respectively.

^c^ Genotypes based on the 14 MST assays in Hornstra *et al.* [28].

^d^ Cb175 is on branch Br.I.002 (ST17) in Hornstra *et al.* [28], but no assay is developed for this branch.

^e^ The genome became available after assay design.

### Synthetic control templates

**Figure 2:**
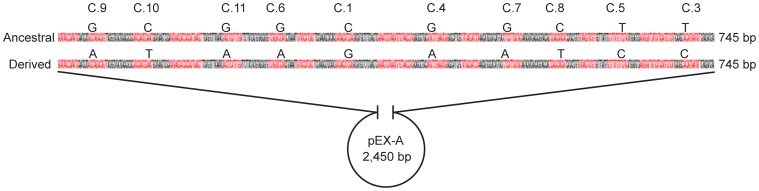
Synthetic vector controls. Two control sequences with ancestral or derived SNP alleles for ten synonymous SNP markers (C.1 to C.11) were synthesized and cloned into the standard vector pEX-A (Fig. S2). Primer binding sites are marked in red, with the SNP states for the markers shown above them.

Two types of synthetic control approaches were tested with the aim of developing an assay that could be performed in laboratories without access to genomic controls. The first approach, where the control sequences were cloned into the pEX-A vector (Fig. 2, Fig. S2), and the second approach, where the control sequences were generated by PCR amplification (Table 3), revealed that both approaches worked as intended. The melt profiles of the synthetic control sequences in both approaches were identical to those for the available genomic DNA and multiple displacement amplification (MDA) control sequences with respect to all of the tested markers. Since we do not possess the Dugway strain, the synthetic controls for the derived state of C.6 could not be compared to genomic DNA. However, the PCR amplified DNA control template for C.6 produced a melt profile that was identical to that of the synthetic vector, with a Tm of 79.8°C.

### Genotyping of isolates

Melt-MAMAs capable of discriminating between the major clades in the tree constructed for the 21 studied *C. burnetii* genomes were developed based on 10 canSNPs. We were able to assign 26 *C. burnetii* isolates, 10 of which had known genome sequences, to one of the tree's clades (Table 4, Fig. 1). All Melt-MAMAs produced melt curves that clearly discriminated between the ancestral and derived SNPs, with ΔTm values ranging from 3.7–5.5°C, while avoiding problems arising from the formation of secondary structures after 40 PCR cycles. In all cases bar one, the results obtained were consistent with genotyping data from previous studies: The synonymous canSNP assays placed our Balaceanu strain in clade C.10 (genomic group I), but MST data from previous investigations suggest that this strain belongs to genomic group II [26].

In several cases, whole genome amplified samples produced using MDA yielded ambiguous melt curves and tended to form double peaks for some markers. In such cases, the MDA samples were replaced with the original genomic DNA preparations, which removed this effect.

### Genotyping of tissue samples

The complete ten assays could be performed on DNA prepared from three authentic tissue samples from Q fever positive goat and sheep placentas sampled in the Netherlands in 2009. The results showed that all three tissue samples were derived for canSNP C.11 (corresponding to MST SNP Cox18bp166 defining the branch Br.II.001 in [28]) (Table 4).

## Discussion

In recent years, several large outbreaks of Q fever have been reported in Europe, clearly demonstrating the need for improved epidemiological tools that can be used to track *C. burnetii* strains in both genetic and geographic terms. Therefore, we have developed a Melt-MAMA SNP typing method targeting SNPs in conserved intragenic regions that can be used in a wide range of laboratories that lack control DNA.

### Genome sequencing

Eight new genomes were sequenced to expand the coverage of the known genetic diversity of *C. burnetii* [45]. This represents a near-doubling in the quantity of publically-available sequence data for this species, and will greatly facilitate future studies on its evolution and provide new signatures that can be used for genotyping. Together with the 13 previously published genome sequences, these new data were used in a phylogenetic analysis of *C. burnetii* in which it was confirmed to have a highly clonal population structure. This implies that canonical SNPs should be very suitable markers for typing this bacterium, with the ability to reveal robust phylogenetic relationships between isolates, as it has previously been proved to be in other highly clonal human pathogenic bacteria such as *Bacillus anthracis* [38], *Yersinia pestis* [36], [37], and *Francisella tularensis* [31]–[35]. In epidemiological investigations, this can facilitate the tracking of the disease source and the geographical origin of outbreak strains in order to develop a strategy for disease prevention and control.

### Typing scheme based on synonymous canonical SNPs

The typing scheme presented in this work is compatible and complementary with the MST scheme developed by Hornstra *et al.* [28]. Both typing schemes use Melt-MAMA to determine the SNP status and the same reagents. Therefore the new markers can easily be integrated with existing MST assays. Each canSNP in this work has a counterpart among the assayed canSNPs in MST regions in all cases but two; C.3 targets the clade starting with branch Br.IV.003 (ST1–4); and C.5 targets the clade starting with branch Br.IV.010 (ST30), and could thereby be used as a complement to the typing scheme presented in Hornstra *et al.* However, unlike MST, the canSNPs here are positioned in regions that have comparably fewer polymorphisms, which can be advantageous because there is a higher likelihood of obtaining PCR products when using primers targeting such regions. We preferred to select synonymous over non-synonymous SNPs to reduce the risk of selecting SNPs that we believe could potentially revert due to an increased selection pressure on the latter ones. This has to be evaluated for *Coxiella* and may not be of any importance for the typing outcome in a highly clonal pathogen as *C. burnetii*. The variation in MST regions was described in Hornstra *et al.* [28] and was confirmed by our analysis of the MST primers and regions in 21 *C. burnetii* genomes, which identified several mutations.

A limitation of the SNPs targeted in this work is that they were selected from a smaller set of isolates than most MST SNPs, and have not yet been tested widely.

The typing scheme and assay presented herein are also compatible with other genotyping technologies, including sequencing and sensitive TaqMan MGB probes, and can be adapted for use in traditional PCR and agarose gel product analyses [43]. Sequence data from new isolates can easily be incorporated into an updated scheme using additional canSNPs. The assays presented herein target 10 major branches of the *C. burnetii* phylogenetic tree, but the method is flexible so new branches and the corresponding canSNPs can be incorporated as additional genomic information becomes available. In addition, the typing resolution can be adjusted to suit the purposes of the study at hand. Finally, incongruences are readily identified using this method because each locus that builds up the genotype carries specific phylogenetic structural information that should be consistent along the analyzed lineage. It is essential to identify incongruences and establish reliable genetic relationships between strains in epidemiological investigations because failing to do so has serious effects on the outcome of the analysis and the predicted dispersal routes. Our analysis of the previously published variable microsatellite (MS) loci (which contain tandem repeats but also a mix of other evolutionary events) in the 21 genomes (Table S1) indicates that the SNP phylogeny could be used as a phylogenetically stable backbone for typing of the more variable MS markers and may help in identifying homoplastic typing results generated by techniques based on the determination of fragment lengths or repeat numbers. It is thus possible that the combined use of canSNPs and MS markers could yield a higher typing resolution than would be achieved with canSNPs alone while also providing more accurate phylogenetic relationships than would be achieved using MS markers alone. In general, the short reads for some genomes (36 bp) did not cause any difficulties in the assembly and extraction of theoretical PCR product sequences in the repeat regions, but in some cases they could not be generated from all of the studied genomes. The product sizes and repeat numbers can then easily be compared with experimental data published by other researchers, but was not investigated further in this work.

### Synthetic control templates

The construction and use of synthetic control templates in this work makes it possible to use the Melt-MAMA SNP typing method even when genomic control DNA collections are not readily available, as is the case for all non-specialized laboratories. The PCR amplified control and the control vector approaches were both viable and provided comparable melt profiles, but the vector approach is preferable to the PCR amplified control because of its simplicity. It is based on the use of two control vectors, one of which carries all of the ancestor SNP marker sequences while the other carries all of the derived SNP marker sequences. The two vectors can thus be used as controls for all of the markers developed in this work and could easily be made available commercially at little cost. This would provide a significant advantage since obtaining genomic DNA from cultures is tedious, expensive and require great experience. The vector format is likely to be more stable than controls generated by PCR and should allow for more accurate determination of copy number concentrations. One limitation of the control vector approach is that as new markers are developed and more genomic information becomes available, it will be necessary to develop and distribute new vectors. However, new marker control sequences can easily be added to the existing control vectors and resynthesized accordingly. Conversely, PCR amplified controls are prepared separately for each marker. Importantly, the method of synthetic controls can be rapidly adapted and expanded to other signatures, and new canSNPs can be included as soon as they are discovered.

### Genotyping of isolates and tissue samples

#### Assignment of isolates to clades

Twenty-six isolates were genotyped using the canSNP assays, yielding results that were consistent with those from the MST SNP assays. The sensitivity and accuracy of Melt-MAMA both seem to be somewhat lower than those achieved with some alternatives such as hydrolysis probe assays [43]. Factors such as high salt concentration and low DNA copy numbers can potentially cause Tm shifts or ambiguous melt profiles, which could potentially lead to incorrect base calls. Although a few isolates occasionally showed Cq-values >33 when typed using the canSNP assays (Cq-values not shown), the overall consistency of the melt profiles, agreement with the melt profiles of the positive controls, and the lack of contradictions, all suggest that the bases were called correctly. In addition, we did not observe any product formation in the no template controls. We aimed to design primers that were not limited to low cycle numbers or prone to secondary product formation, even in samples with more complex biological backgrounds, as infected tissue. The Melt-MAMA method is highly sensitive to secondary product formation because it is based on comparative analyses of the dissociation curves for small amplicons. All of the developed canSNP assays developed here use 40 PCR cycles, while the previously published MST assays use 30–33 PCR cycles, making the former more sensitive. This could be because of higher probability to find target regions optimal for primer design when using whole genomes compared to the limited number of targets using MST regions only.

#### Multiple displacement amplification (MDA) occasionally gives double peaks

Unexpectedly, we obtained ambiguous melting profiles in the form of double peaks when genotyping some of the MDA templates. This disappeared when the original genomic DNA preparation was used. We speculate that the ambiguous results were due to contaminants that were co-amplified and favored during the whole genome amplification step.

#### Direct genotyping of *Coxiella* in total DNA preparations from cell cultures and tissues is possible

The results presented herein demonstrate that genome sequencing of total DNA preparations from cell cultures containing as little as 6% *Coxiella* DNA (strain 2338) can yield sufficient coverage for a draft genome sequence that could be used for the theoretical identification of canSNPs. This result is consistent with the theoretical value where genome sequences currently could be assembled from DNA preparations containing as little as 0.1% *Coxiella* DNA using a single lane of the HiSeq2000 instrument (Illumina, Hayward, CA, USA). Further, the material could also be used for genotyping, indicating that the developed method is suitable for both direct use of total DNA isolated from possibly contaminated samples as well as pure genomic *Coxiella* DNA preparations. This was confirmed by genotyping DNA extracted from tissue samples from three infected Dutch sheep and goats using the newly developed canSNP assays. This demonstrated the usefulness of the developed assays for direct genotyping of *Coxiella* in total DNA preparations also from tissues.

## Conclusions

The eight new genomes, the typing scheme based on canSNPs, and the synthetic control templates developed in this study all increased the reliability and quality of existing phylogenetic analyses of *C. burnetii*, confirmed its highly clonal population structure, and provides a complement to existing genotyping methods.

Laboratories lacking control DNA can now perform Melt-MAMA SNP typing of *C. burnetii* isolates and tissue samples at low cost and with typing results that are directly comparable across laboratories. Additional research is required to further validate the canonical properties of the selected SNPs and the sequence identity of the primers. However, because accurate genotyping, assay accessibility and inter-laboratory comparisons are all most important for rapid and accurate epidemiological source tracking, the results presented herein represent a significant and promising step forward in the monitoring and analysis of bacterial disease outbreaks.

## Materials and Methods

### Isolates and tissue samples

Twenty-six *C. burnetii* isolates and three samples from infected goat or sheep placentas from the Netherlands were selected to represent the known diversity of *C. burnetii* (Table 1). However, representatives for genomic group III were lacking in this collection. Eight of the 26 isolates (Scurry, C2, S4, Z349-36/94, Ohio, Innsbruck, 2338, Namibia), were subjected to genome sequencing. These eight isolates and two additional isolates (Nine Mile RSA493 and Priscilla Q177) that have published genome sequences were used to evaluate the performance of the developed assay and the properties of the selected SNPs.

### Ethics statement

Placenta specimens were collected by a veterinarian on (putative) Q fever positive farms for initial testing at the Animal Health Service (GD, Deventer) as part the Dutch Q fever monitoring program since Q fever is a notifiable disease in the Netherlands both for humans and animals. No invasive methods were used whatsoever and all obtained placenta material was excreted as part of the natural delivery process of the animal. Approval from an ethics committee was for that reason not sought after.

### Cultivation

Cells from the BGM line (Flow Laboratories, Rockville, MD, USA) were grown in Eagles minimal essential medium (MEM) supplemented with Earls salts, 2 mM L-glutamax 5%; Fetal Calf Serum (FCS); 1% Non-Essential Amino acids (NEA); 0.2% sodium bicarbonate (NaHCO_3_) (Sigma-Aldrich, St Louis, MO, USA). Confluent cell layers were infected with bacteria and incubated at 37°C. Fresh media was added after 20–24 h. To enhance vacuole formation, the infected confluent BGM cells were divided using trypsin. *C. burnetii* were collected from the media of actively growing cultures after 7–8 days by differential centrifugation. An initial centrifugation was performed at 500×g (1,500 rpm) for 5 minutes at 4°C to remove cell debris, followed by a second centrifugation at 2,550×g (3,500 rpm) for one hour at 4°C to collect the bacteria.

### Preparation of DNA from cell cultures

The bacteria from the confluent growing cell cultures were harvested by differential centrifugation, as decribed above. The bacteria were washed twice in PBS. The bacterial pellet was then resuspended in 50 mM Tris (pH 7.8) and mixed with 10 mM MgSO_4_ solution containing 20 µg DNase (Ambion, Life Technologies, Carlsbad, CA, USA). The resulting suspension was incubated at 37°C for 30 minutes. 0.5% SDS and 50 µg/ml proteinase K solution were then added and the sample was incubated at 56°C for one hour. After cooling to room temperature, 100 mM Tris (pH 7.8), 1 mM EDTA, a 15% sucrose solution, and 1 mg/ml lysozyme solution (Sigma-Aldrich) were added and the resulting mixture was incubated at 37°C for 16 h. On the following day, 100 mM Tris (pH 12.0), 1 mM EDTA, and 5% SDS were added and the sample was incubated at 56°C for one hour.

The sample was then cooled to room temperature and treated with phenol/chloroform twice before three volumes of ice cold (−20°C) 99.5% ethanol to precipitate the DNA, was added. After incubation at −20°C for 30 min, the sample was centrifuged at 19,000×g (15,000 rpm) for 30 min. The pellet was then resuspended in 1×TE containing 50 µg RNase (Epicentre, Madison, WI, USA) and incubated at 37°C for one hour. Proteinase K (500 µg, Epicentre) was then added and the resulting mixture was incubated for another hour at 37°C. The sample was treated with phenol/chloroform twice before precipitation of the DNA by adding 0.1 volume of 3 M sodium acetate and 2.5 volumes of ice cold (−20°C) 99.5% ethanol. The resulting mixture was then incubated at −20°C for 30 min, centrifuged, and washed twice with 80% ethanol. After centrifugation the pellet was air dried and resuspended in 1×TE.

### Preparation of DNA from goat and sheep placentas

A small piece of material was transferred to a screw cap tube containing a glass bead (5 mm) and 500 µl of a 0.9% sodium chloride solution. The material was homogenized by bead beating for 4×30 s (Retsch MM400, Haan, Germany) at a frequency of 30 s^−1^, after which it was processed using the MasterPure™ DNA Purification kit (Epicentre Biotechnologies, Madison, WI, USA) according to the instructions supplied by the manufacturer.

### Genomic DNA amplification

Low concentration DNA samples were amplified using two different Multiple Displacement Amplification (MDA) kits: the Illustra GenomePhi V2 Amplification Kit (GE Healthcare Life Sciences, Uppsala, Sweden) and the REPLI-g UltraFast Mini Kit (Qiagen, Hilden, Germany). In both cases, the amplifications were performed according to the manufacturers' instructions. Once the amplifications were complete, the enzymes were inactivated by heating the samples to 65°C. The amplified DNA was then diluted with sterile distilled water to facilitate subsequent DNA extraction using phenol/chloroform. The DNA was precipitated using 0.1 volumes of 3 M sodium acetate and 2.5 volumes of ice cold (−20°C) 99.5% ethanol followed by incubation for 16 h at −20°C. The precipitated samples were centrifuged at 19,000×g (15,000 rpm) for 30 minutes at 4°C, washed once with 70% ethanol and air dried. The pellet was then dissolved in 1×TE and the DNA concentration was estimated using a Qubit fluorometer (Life Technologies, Carlsbad, CA, USA).

### Genome sequencing and assembly

DNA from cell cultures was sequenced using different generations of the Illumina (Illumina, Hayward, CA, USA) technology (GenomeAnalyzer II single-end reads of 36 bp/71 bp, HiSeq2000 pair-end reads of 100 bp and MiSeq paired-end reads of 150 bp) according to the manufacturer's instructions. Sequence reads were mapped to the reference genome Nine Mile RSA493 (GenBank AE016828.2) using Bowtie2 [46] and the coverage statistics were calculated using BEDTools [47]. The percentages of *Coxiella* reads in the new genomes were estimated by using Bowtie2 [46] to align the reads against all published *Coxiella* genome and plasmid sequences (Table 1); the results obtained were 48.9% for Scurry, 93.5% for C2, 96.7% for S4, 49.1% for Z349-36/94, 55.2% for Ohio, 85.9% for Innsbruck, 6.6% for strain 2338 and 9.0% for Namibia. Sequence reads were filtered to remove host cell reads by aligning the reads against the *Cholocebus aethiops* draft genome (NCBI BioProject accession PRJNA168621) [48]. The mean sequence depth across the Nine Mile RSA493 reference genome (GenBank AE016828.2) were between 86× and 1063× and more than 97% of the genomes had 10× coverage or greater (Table 2). All of the generated sequence data were deposited with the NCBI BioProjects accession PRJNA197120 and PRJNA197122 to PRJNA197128.

The phylogenetic analysis of the 8 new and 13 published genomes was performed using the neighbor joining clustering method computing the number of base differences per sequence with the Nine Mile RSA493 strain as a reference. All positions containing gaps and missing data were eliminated. The tree was rooted according to Pearson *et al.* 2013 [49] (Fig. 1).

### *In silico* analysis of MLVA loci

*In silico* PCR product sequences in 21 genomes were generated for 17 variable regions from primer sequences described in Arricau-Bouvery (MS01, 03, 07, 12, 20–24, 26, 30, 31, 33, 34, 36) [23] and Roest (MS27, 28) [2] using an in-house Python pipeline that utilizes Multithreaded Electronic PCR [50].

### Selection of canSNPs and design of primers

Biallelic SNPs within coding regions were selected from a multiple alignment of 18 *C. burnetii* genomes calculated using the progressive Mauve algorithm [51]. The genomes Cb109, Cb175 and Z3055 were not published at the time of assay design and were therefore not included. All positions within 30-bp of gaps (“-”) or uncertain positions (“n”) were excluded to minimize potential misalignment errors. CanSNPs were identified using the VariantClassifier program with the strain Nine Mile RSA493 as a reference [52]. Initially, several putative SNPs were chosen to define ten markers that resolved the major phylogenetic branches depicted in Figure 1. Of these, one representative SNP was selected for each marker that provided the most optimal primer design and assay characteristics. The primers and product regions contained no polymorphisms other than the selected canSNPs in the 21 genomes. SNP states were distinguished based on their differential melting profiles using the Mismatch Amplification Mutation Assay (Melt-MAMA). Primers were designed according to the guidelines [43], [53]. In brief, each marker assay included two SNP-specific competing forward primers and one reverse common primer (Table 3). Allele specificity was assigned to the ultimate nucleotide at the 3′ end of each forward primer and a mismatch was introduced in the antepenultimate nucleotide. In order to distinguish between amplicon melt profiles, a GC-rich tail was incorporated at the 5′ end of the forward primer defining the derived allele state. Primers were evaluated and analyzed for secondary structures using AlleleID software (v.7.7, Premier Biosoft International, Palo Alto, CA, USA). All primers were ordered from Eurofins MWG operon (Ebersberg, Germany).

### Synthetic positive controls

To address the challenges associated with the limited availability of genomic control DNA in most laboratories, we evaluated synthetic control sequences cloned into a standard vector, pEX-A (F- *mcr* A Δ(*mrr-hsd* RMS-*mcr* BC) Φ 80*lac* ZΔM15 Δ*lac* X74 *rec* A1 *ara* D139 Δ(*ara-leu*)7697 *gal* U *gal* K *rps* L (Str^R^) *end* A1 *nup*G, vector size 2,450 bp). Two vectors were synthesized with a 745 bp insert containing the ancestral and derived SNP alleles for all ten synonymous SNP markers, respectively (Fig. 2, complete sequences in Fig. S2). Readymade vectors were ordered from Eurofins MWG operon, Ebersberg, Germany. The control vectors were evaluated at a concentration of 10^6^ copies/reaction, as determined using a Qubit fluorometer (Life Technologies, Carlsbad, CA, USA), by comparing their melt profiles to those for 1 ng/reaction of genomic DNA or MDA templates of known genotypes.

Given the lack of a suitable control for the derived state of marker C.6, PCR-amplified synthetic controls were evaluated as an alternative to the vector-based synthetic controls. The PCR-amplified control template for the derived SNP state of C.6 was synthesized from an ancestral template using the control primers found in Table 3 (adopted from Birdsell *et al.* 2012). Approximately, 1 ng of ancestral template (Priscilla Q177) was amplified using 200 nM of forward (C.6 Der Cont.) and reverse primer (C.6 Com Cont.) in a total reaction volume of 10 µl, under the same reaction conditions as previously described. The product was diluted by a factor of 10^−6^ and 1 µl of the resulting solution was used in the control reactions.

### Quantitative real time PCR

PCR amplifications were performed using 1×SYBR® Green PCR Master Mix (Applied Biosystems, Life Technologies, Carlsbad, CA, USA), two competing forward primers and one common reverse primer with optimized concentrations as shown in Table 3 (200 nM was set as the default primer concentration), in a total volume of 10 µl [28], [32]. The primer concentrations were optimized to provide unambiguous melt curves due to occasional amplifications of the mismatched competing primers. Forward primers with lower efficiencies than their competing primers were titrated until clear-cut melt curves were obtained. The assay for the C.6 marker required the addition of a small GC-tail to the common reverse primer because the product GC content was biased, resulting in internal Tm variation and split melt peaks.

Approximately 1 ng of DNA template was added to each reaction, measured using a Qubit fluorometer (Invitrogen, Carlsbad, CA, USA). The DNA concentration did not necessarily reflect the true amount of *C. burnetii* DNA template, since residual BGM cell line DNA from the culture step may lead to overestimation of the concentration. The reaction was cycled with a CFX96™ Real-time detection system (Bio-Rad Laboratories, Hercules, CA, USA) using a thermal cycle consisting of an initial denaturation and enzyme activation step at 95°C for 10 min followed by 40 cycles at 95°C for 15 s and 60°C for 60 s. Fluorescence melt curve analysis was performed at 65°C–85°C with a 5 s/0.2°C increment. The data were processed and analyzed using the Bio-Rad CFX manager. For comparison, MST SNP assays were run in parallel as described in Hornstra *et al.* [28].

### Genotyping of isolates

A set of twenty six *C. burnetii* isolates, including 10 published and herein genome sequenced isolates, were genotyped in duplicate using the developed Melt-MAMAs, together with positive and no template controls (Table 1). The assay performance and the properties of the canSNPs were evaluated (Table 3, Table 4). A number of the templates in the set were whole genome amplified as described above. In addition, the original DNA preparations were replaced with MDA templates in some cases, because only limited quantities of the relevant DNA were available. MDA templates that yielded obscure melt profiles were reanalyzed using the original DNA preparations.

### Genotyping of tissue samples

Samples from three infected placentas were genotyped with the 10 canSNP Melt-MAMAs. The extracted DNA was diluted 10^−2^–10^−3^–fold and 1 µl of the resulting solution was added to each reaction, with a total reaction volume of 10 µl. All analyses were performed in duplicate and all samples were analyzed alongside positive and template-free controls.

## Supporting Information

Figure S1 **Incidence of Q fever in the Netherlands 2009.** The three Q fever-positive placentas analysed in this study were sampled in the south of the Netherlands where the Q fever epidemic was most predominant. The spots where the three placentas were collected are not shown with respect to the owners of the farms.
(PDF)

Figure S2 **Ancestral and derived control vector insertion sequences.** Primer binding sites are marked with boxes. CanSNPs are shown in boldface. Marker control sequences are placed in the following order: C.9, C.10, C.11, C.6, C.1, C.4, C.7, C.8, C.5 and C.3, but in variable directions. Inserts were cloned into a pEX-A vector, total size 3,195 bp (2,450 bp+745 bp). See also Figure 2.
(PDF)

Table S1 ***In silico*** **microsatellite (MS) analysis.** Sequence lengths of 17 variable MS loci and the calculated number of repeats in 21 genomes. Homoplasy was observed in seven out of 17 investigated MS loci.
(XLS)
