# Supplementary figures and images for: Eight New Genomes and Synthetic Controls Increase the Accessibility of Rapid Melt-MAMA SNP Typing of Coxiella burnetii
Source: PLoS One. 2014 Jan 21;9(1):e85417. doi: 10.1371/journal.pone.0085417 (PMC3897454; doi:10.1371/journal.pone.0085417)

# Incidence per county

Number per 100.000 inhabitants

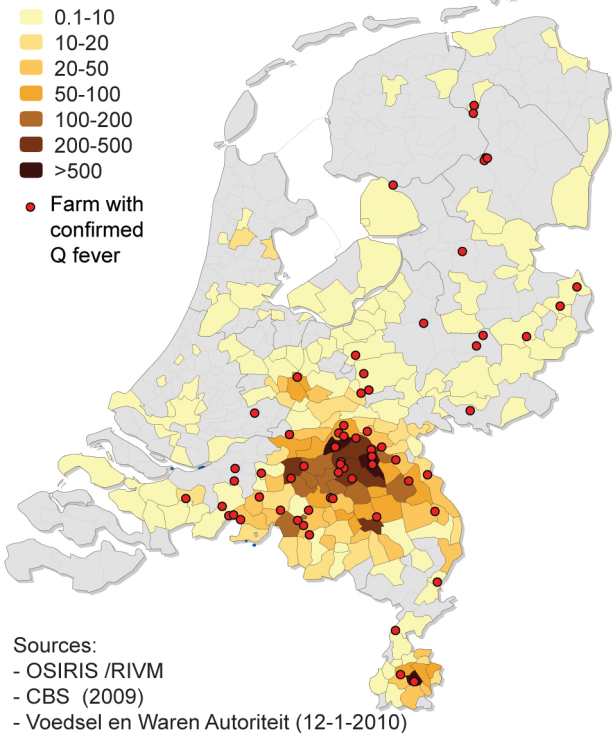

Supplement: Figure S1 — Incidence of Q fever in the Netherlands 2009. The three Q fever-positive placentas analysed in this study were sampled in the south of the Netherlands where the Q fever epidemic was most predominant. The spots where the three placentas were collected are not shown with respect to the owners of the farms. (PDF) [file pone.0085417.s001.pdf]
