# Supplementary material for: Eight New Genomes and Synthetic Controls Increase the Accessibility of Rapid Melt-MAMA SNP Typing of Coxiella burnetii
Source: PLoS One. 2014 Jan 21;9(1):e85417. doi: 10.1371/journal.pone.0085417 (PMC3897454; doi:10.1371/journal.pone.0085417)
Supplement: Figure S2 — Ancestral and derived control vector insertion sequences. Primer binding sites are marked with boxes. CanSNPs are shown in boldface. Marker control sequences are placed in the following order: C.9, C.10, C.11, C.6, C.1, C.4, C.7, C.8, C.5 and C.3, but in variable directions. Inserts were cloned into a pEX-A vector, total size 3,195 bp (2,450 bp+745 bp). See also Figure 2. (PDF) [file pone.0085417.s002.pdf]

Figure S2. Ancestral (A) and Derived (B) Control vector insertion sequences. Primer binding sites are marked with boxes. CanSNPs are shown in boldface. Marker control sequences are placed in the following order: C.9, C.10, C.11, C.6, C.1, C.4, C.7, C.8, C.5 and C.3, but in variable directions. Inserts were cloned into a pEX-A vector, total size 3,195 bp (2,450 bp + 745 bp). See also Figure 2.

A. Ancestral Control (745 bp)

GAGATATATAACCCCAATAATTGCCAAATCGCATC**G**ACGCCTAAGCAACCAGGTTTTACGGGGTCATGACTGCAA

CACCGCCGCCAATACCCAACATGCCAAAACT**C**GGCATGAAAGCTTCCGCAACAATGAAAGCCAATCCCACCCAAC

TTGAAACGCTTTTTCGATGGTGTTTTTATAAATATC**G**TAACCTGTCCATTTGTTGGGATTTTCTTTTATCGTACT

GGTGAGGCAGGTGGTTCGTAGGGATCGCTAAT**G**CCTGTAAAAATTTTGATTATACCCATTCGAGCTTTTCCGTAA

AGACGCTCATCATACGCAATCAAACCATCTCGCAA**C**GATCGATTGGCTTCTTTCTGAAGCTTCGAAGAAATCGTC

GTATAAACATTGTTGCTTAAAGCCCGTACGACAGGCGCTTGACC**G**ATGACTTCTTGGAAACATCGAGGACGCCAT

TTTCGTGCTTATTCAACGCAATCTCCTAAAAACCAGTGCGCGATCCATGTTGAC**G**TCTTCCCATTTTCGACTGAC

CAAAGTACAGATCGAACCACGGGTAACCCAGCTCGCAACACCATACCAAT**C**CCATCACCTGTGTTGGTATAAGCG

TTACTGGTTGGGTTTTTAATTGTTGCTGTTTTATTGTTGTTCGTTAG**T**ATTGGGAGTGTCGAAGATGGGAGTTTG

ATTGCATTCACAATGTTGCTGCTATTGCACAGGC**T**CAACCAGGTGAAATTAGTTTCGTAACGGATCGTAA

B. Derived Control (745 bp)

GAGATATATAACCCCAATAATTGCCAAATCGCATC**A**ACGCCTAAGCAACCAGGTTTTACGGGGTCATGACTGCAA

CACCGCCGCCAATACCCAACATGCCAAAACT**T**GGCATGAAAGCTTCCGCAACAATGAAAGCCAATCCCACCCAAC

TTGAAACGCTTTTTCGATGGTGTTTTTATAAATATC**A**TAACCTGTCCATTTGTTGGGATTTTCTTTTATCGTACT

GGTGAGGCAGGTGGTTCGTAGGGATCGCTAAT**A**CCTGTAAAAATTTTGATTATACCCATTCGAGCTTTTCCGTAA

AGACGCTCATCATACGCAATCAAACCATCTCGCAA**G**GATCGATTGGCTTCTTTCTGAAGCTTCGAAGAAATCGTC

GTATAAACATTGTTGCTTAAAGCCCGTACGACAGGCGCTTGACC**A**ATGACTTCTTGGAAACATCGAGGACGCCAT

TTTCGTGCTTATTCAACGCAATCTCCTAAAAACCAGTGCGCGATCCATGTTGAC**A**TCTTCCCATTTTCGACTGAC

CAAAGTACAGATCGAACCACGGGTAACCCAGCTCGCAACACCATACCAAT**T**CCATCACCTGTGTTGGTATAAGCG

TTACTGGTTGGGTTTTTAATTGTTGCTGTTTTATTGTTGTTCGTTAG**C**ATTGGGAGTGTCGAAGATGGGAGTTTG

ATTGCATTCACAATGTTGCTGCTATTGCACAGGC**C**CAACCAGGTGAAATTAGTTTCGTAACGGATCGTAA
